# Dissecting out the Complex Ca^2+^-Mediated Phenylephrine-Induced Contractions of Mouse Aortic Segments

**DOI:** 10.1371/journal.pone.0121634

**Published:** 2015-03-24

**Authors:** Paul Fransen, Cor E. Van Hove, Arthur J. A. Leloup, Wim Martinet, Guido R. Y. De Meyer, Katrien Lemmens, Hidde Bult, Dorien M. Schrijvers

**Affiliations:** 1 Department of Pharmaceutical Sciences, University of Antwerp, Antwerp, Belgium; 2 Department of Medicine and Health Sciences, University of Antwerp, Antwerp, Belgium; Cinvestav-IPN, MEXICO

## Abstract

L-type Ca^2+^ channel (VGCC) mediated Ca^2+^ influx in vascular smooth muscle cells (VSMC) contributes to the functional properties of large arteries in arterial stiffening and central blood pressure regulation. How this influx relates to steady-state contractions elicited by α1-adrenoreceptor stimulation and how it is modulated by small variations in resting membrane potential (V_m_) of VSMC is not clear yet. Here, we show that α1-adrenoreceptor stimulation of aortic segments of C57Bl6 mice with phenylephrine (PE) causes phasic and tonic contractions. By studying the relationship between Ca^2+^ mobilisation and isometric tension, it was found that the phasic contraction was due to intracellular Ca^2+^ release and the tonic contraction determined by Ca^2+^ influx. The latter component involves both Ca^2+^ influx via VGCC and via non-selective cation channels (NSCC). Influx via VGCC occurs only within the window voltage range of the channel. Modulation of this window Ca^2+^ influx by small variations of the VSMC V_m_ causes substantial effects on the contractile performance of aortic segments. The relative contribution of VGCC and NSCC to the contraction by α1-adrenoceptor stimulation could be manipulated by increasing intracellular Ca^2+^ release from non-contractile sarcoplasmic reticulum Ca^2+^ stores. Results of this study point to a complex interactions between α1-adrenoceptor-mediated VSMC contractile performance and Ca^2+^ release form contractile or non-contractile Ca^2+^ stores with concomitant Ca^2+^ influx. Given the importance of VGCC and their blockers in arterial stiffening and hypertension, they further point toward an additional role of NSCC (and NSCC blockers) herein.

## Introduction

Arterial stiffness and hypertension are common clinical conditions in the elderly that lead to increased cardiovascular risk. In the isolated aorta, in animal models of endothelial dysfunction and in humans, reduced nitric oxide (NO) bioavailability induces physiological alterations including arterial stiffness, vascular wall remodeling and hypertension [1,2]. Thereby, arterial smooth muscle cells in cross-talk with endothelial cells, determine arterial mechanics [3], including the basal tonus of the blood vessel. Both L-type Ca^2+^ channels blockers and basal NO release from elastic arteries reduce arterial stiffness [4]. However, the specific role of L-type Ca^2+^ influx in arterial (de-)stiffening needs further investigation.

Isolated vascular smooth muscle cells (VSMC) display a window voltage range, in which a “time-independent” L-type Ca^2+^ influx or window Ca^2+^ current flows [5–8]. The physiological significance of this background window Ca^2+^ influx in multicellular preparations, and more specifically, in mouse aortic segments was evident in segments depolarised with elevated extracellular K^+^ [9–11]. How it contributes to contractions induced by α_1_-adrenoceptor stimulation is not clear. Elevated extracellular K^+^ and α_1_-adrenoceptor stimulation of VSMC causes contraction initially by intracellular Ca^2+^ release via IP_3_-receptor-mediated Ca^2+^ release from sarcoplasmic reticulum (SR) Ca^2+^ stores, followed by Ca^2+^ influx via Ca^2+^ permeable ion channels and concomitant Ca^2+^ sensitization [12–14]. Yet, even these contractions are associated with depolarisation [15–19]. Therefore, it is hypothesized that α_1_-adrenoceptor-stimulation with PE induces IP_3_-mediated intracellular Ca^2+^ release, activates non-selective cation channels (NSCC), causes depolarization, opens L-type Ca^2+^ channels (VGCC) with concomitant background window Ca^2+^ influx and elicits contraction. The L-type Ca^2+^ influx is not only important for contraction of mouse aortic segments but also for their relaxation by endothelium-derived factors. Indeed, the relaxing efficacy of NO is dependent on the amount of Ca^2+^ influx via L-type Ca^2+^ channels [19].

In the present study, aortic segments of C57Bl6 mice were α_1_-adrenoceptor stimulated with phenylephrine (PE) to investigate the involvement of NSCC and VGCC Ca^2+^ influx in the isometric contraction, and more specifically, the potential role of the VGCC window Ca^2+^ influx herein. The relationship between Ca^2+^ mobilisation and isometric tension revealed that the contraction induced by intracellular Ca^2+^ release with PE could be isolated from the contraction evoked by Ca^2+^ influx. The latter, tonic contractile component involved both Ca^2+^ influx within the window voltage range of the VGCC and Ca^2+^ influx via NSCC. Modulation of the window Ca^2+^ influx by small variations of V_m_ of the VSMC caused significant effects on the contractile properties of aortic segments. Results of this study extend our knowledge of the important role of VGCC and NSCC (and their blockers) in arterial stiffening and hypertension, which is of major importance to develop therapeutical strategies for the treatment of arterial stiffness, hypertension and closely associated cardiovascular risk [4,20,21].

## Material and Methods

### Aortic segments

Ethics statement: The studies were approved by the Ethical Committee of the University of Antwerp, and the investigations conform to the Guide for the Care and Use of Laboratory Animals published by the US National Institutes of Health (NIH Publication No. 85–23, revised 1996).

C57Bl6 mice (n = 100, 80 males, 20 females, food and water ad libitum, 12/12 light-dark cycle) were used at the age of 4 to 7 months. Animals were euthanized by perforating the diaphragm under anaesthesia (sodium pentobarbital, 75 mg kg^-1^, i.p.). The thoracic aorta was carefully removed, stripped of adherent tissue and dissected systematically. Starting at the diaphragm, the ascending thoracic aorta was cut in 5 to 6 segments of 2 mm width. Vessels were immersed in Krebs Ringer (KR) solution (37°C, 95% O_2_/5% CO_2_, pH 7.4) containing (in mM): NaCl 118, KCl 4.7, CaCl_2_ 2.5, KH_2_PO_4_ 1.2, MgSO_4_ 1.2, NaHCO_3_ 25, CaEDTA 0.025 and glucose 11.1. High K^+^-solution was prepared as KR solution, but NaCl was replaced with equimolar KCl. When Ca^2+^ was omitted from the KR solution, 1 mM EGTA was added (further named 0Ca) and, hence, to restore 2.5 mM free Ca^2+^ KR solution again, 3.5 mM Ca^2+^ was added to the Ca^2+^- free KR from a 1.75 M CaCl_2_ stock solution (further named +Ca). In the absence of external Ca^2+^, the PE-induced contraction was transient and was called phasic contraction. The contraction upon re-admission of Ca^2+^ was permanent and was called tonic contraction. To avoid spontaneous emptying of the SR stores in 0Ca, PE was always added at 3 minutes after removal of external Ca^2+^. To obtain 0Ca KR solution with different K^+^ concentrations, NaCl in the Ca^2+^-free KR was replaced with equimolar amounts of K^+^. Genders were randomly distributed over the experimental groups. In all experiments where both genders were used, there was no evidence for an influence of gender on the results.

Phenylephrine (PE) was added in concentrations of 1 μM. Extracellular K^+^ was used to clamp the aortic segments at certain potentials [9].

### Isometric tension measurements

Aortic segments were mounted in 10 ml organ baths as described before [19]. Isometric force was acquired at 10 Hz and was reported in mN. To avoid any vasomotor interference due to prostanoids, 10 μM indomethacin was present in all experiments. Endothelial cells were always present but the basal NO formation was inhibited by adding a combination of 300 μM *N* ^*Ω*^-nitro-L-arginine methyl ester (L-NAME) and 300 μM *N* ^*Ω*^-nitro-L-arginine (L-NNA).

### Combined assay of isometric tension and VSMC Ca^2+^

In myograph experiments, the endothelium was mechanically removed by rubbing the interior of the segment with a braided silk wax. Removal of the endothelium in these experiments was necessary to avoid interference of endothelial with VSMC Ca^2+^ signals [19]. The Fura-2 AM (10 μM)-loaded segment was continuously perfused with KR (37°C) which was aerated with 95% O_2_/5% CO_2_ (pH 7.4). The single emission (510 nm) ratio at dual excitation (340 and 380 nm) was used as a relative measure of free [Ca^2+^]_i_ (relative units, RU) and was analysed with Felix software (PTI, USA). Tension was measured simultaneously, acquired at 1 Hz and reported in mN mm^-1^ [19].

### Data analysis

All results are expressed as mean±sem with n representing the number of mice. Time-force curves were fitted with a bi-exponential function revealing amplitudes and time constants of first (fast) and second (slow) components. *Y* = *Y*_0_ + ((*A_fast_* − *Y*_0_) * (1 − exp(− *K_fast_* * (*X* − *X*_0_)) + ((*A_slow_*) − *Y*_0_) * (1 − exp(− *K_fast_* * (*X* − *X*_0_)) with Y_0_, the start amplitude, usually 0 mN; A_fast_, the amplitude and K_fast_, the rate constant of the fast component (K_fast_ = 1/τ_fast_); A_slow_, the amplitude and K_slow_, the rate constant of the slow component (K_slow_ = 1/τ_slow_); X_0_, the time at which force increases.

Because tonic contractions by PE tended to increase for long (>15 minutes) periods of time, the contraction, measured at 650 s was considered as “steady-state” contraction. Concentration-response curves were fitted with sigmoidal concentration-response equations with variable slope, which revealed maximal responses (E_max_) and the logarithm of the concentration resulting in 50% of the maximal excitatory or inhibitory effect (EC_50_ or IC_50_) for each vessel segment. A two-way ANOVA with Bonferroni post test (concentration-response curves) and paired or unpaired *t*-test (GraphPad Prism) were used to compare means of the different experimental groups. A 5% level of significance was selected.

### Materials

Sodium pentobarbital was obtained from Sanofi (Brussels, Belgium), indomethacin from CERTA (Belgium), *N* ^Ω^-nitro-L-arginine methyl ester (L-NAME), *N* ^Ω^-nitro-L-arginine (L-NNA) and nifedipine from Sigma (Bornem, Belgium), Fura 2-AM from Molecular Probes (Invitrogen, Merelbeke, Belgium), 2-aminoethoxydiphenyl borate (2-APB), BAY K8644, levcromakalim, glibenclamide, cyclopiazonic acid (CPA), diltiazem, verapamil from TOCRIS (Bristol, United Kingdom).

## Results

### PE-induced contractions

**Fig 1:**
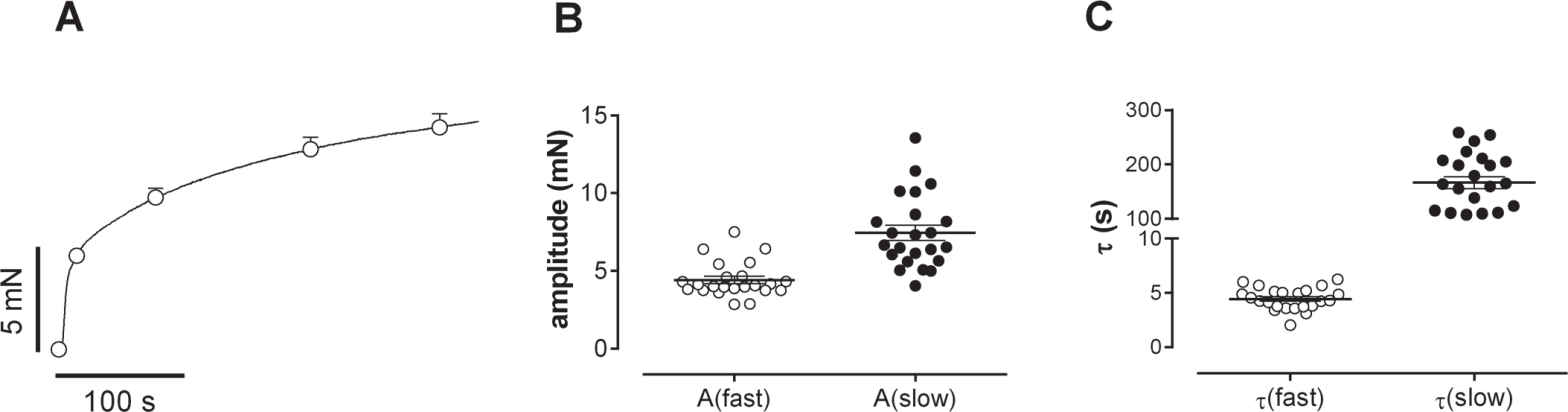
Kinetic analysis of the isometric contractions of mouse aortic segments by 1 μM PE. Aortic segments mounted in organ baths produced isometric contractions as shown in (A). Bi-exponential fits of the contractions in A revealed amplitudes (B) and time constants (C) of fast and slow components. Data were acquired at 10 Hz, specific data points (open circles) are shown as mean± sem (n = 23).

In the organ bath, 1 μM PE caused isometric contractions, which followed a bi-exponential time course (Fig. 1, [22]). Besides a fast phase of contraction with an amplitude of 4.43 ± 0.42 mN (37.7±0.6% of total contraction) and a time constant of 4.4 ± 0.2 s, there was a slow phase of contraction with an amplitude of 7.47±0.49 mN (62.3±0.6% of total contraction) and a time constant of 161±11 s.

**Fig 2:**
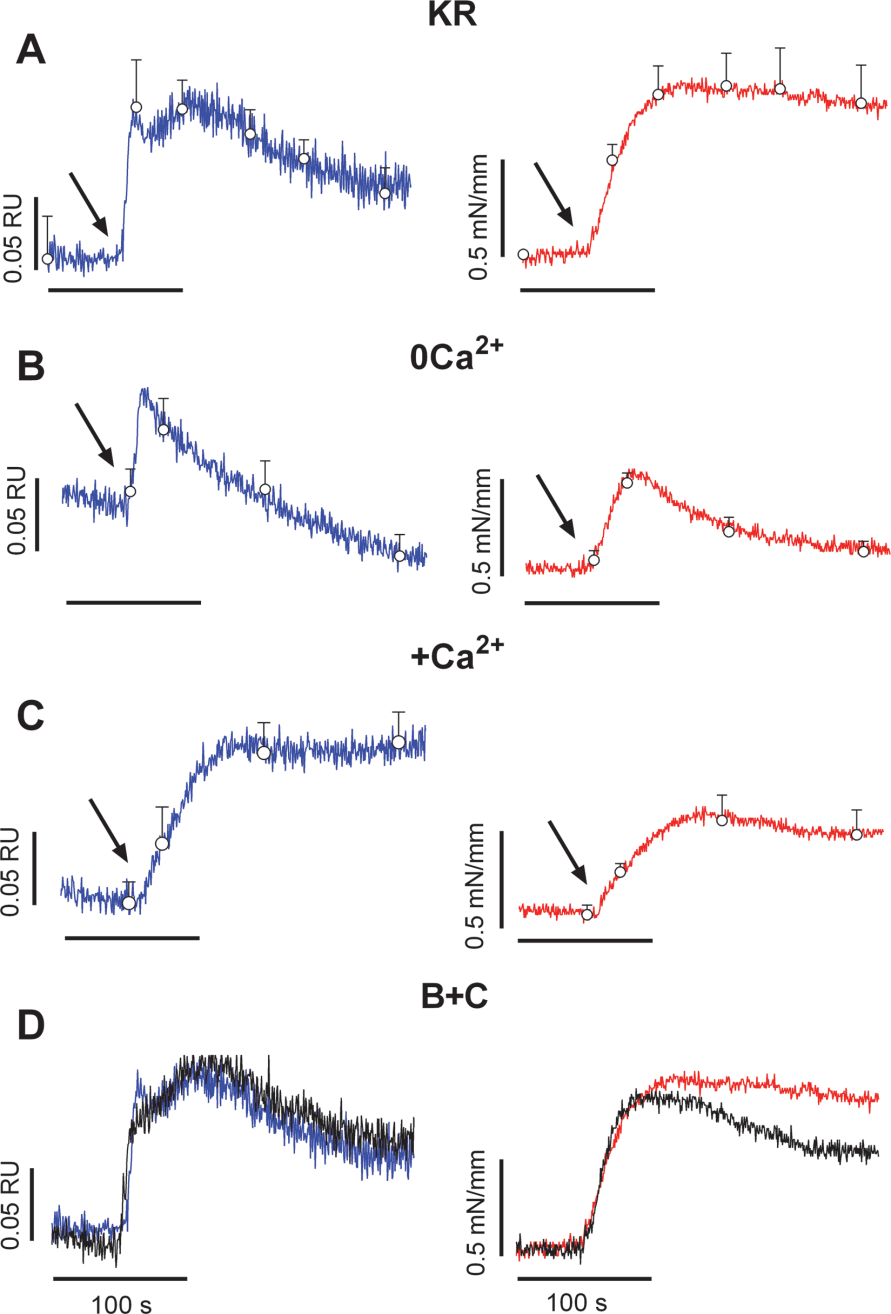
Temporal relationship between intracellular Ca^2+^ signal and isometric contraction by PE. Ca^2+^signal (left, blue) and corresponding isometric tension development (right, red) induced by 1 μM PE (arrow indicates addition) in myograph-mounted endothelium-denuded mouse aortic segments in normal KR solution (A, KR), in Ca^2+^-free KR (B, 0Ca^2+^) and upon re-addition of 3.5 mM Ca^2+^ to the Ca^2+^-free KR solution containing 1 μM PE (C, +Ca^2+^). (D) Sum (B+C) of PE-responses in the absence of Ca^2+^ and upon re-admission of 3.5 mM Ca^2+^ (black) compared with the [Ca^2+^]_i_ and tension signals by PE in control (blue and red). Data were acquired at 1 Hz, some data points (open circles) are shown as mean± sem (n = 6).

To correlate the fast and slow force components with intracellular Ca^2+^ signals in the VSMC, Ca^2+^ and isometric tension were measured simultaneously in endothelium-denuded aortic segments. The addition of 1 μM PE increased both intracellular Ca^2+^ and tension, but the temporal relationship between both parameters was complex (Fig. 2A). Ca^2+^ showed an initial rapid spike followed by a slower increase and then a decrease to plateau values above baseline values. Tension increased to plateau values and then slightly decreased.

Removal of external Ca^2+^ (0Ca) caused a significant decrease of basal [Ca^2+^]_i_ from 0.91±0.03 to 0.81±0.02 RU (p<0.005, n = 6) and of basal tension from 0.52±0.02 to 0.40±0.05 mN/mm (p<0.05, n = 6). Application of 1 μM PE in 0Ca induced transient parallel [Ca^2+^]_i_ and isometric tension increase (Fig. 2B). The peak [Ca^2+^]_i_ was 0.19±0.06 RU and of similar magnitude as in the presence of external Ca^2+^ i.e. 0.17±0.04 RU (p>0.05). Peak PE-elicited tension of 0.72±0.09 mN/mm was significantly decreased compared with the peak tension of 1.13±0.15 mN/mm (p<0.05) in the presence of Ca^2+^.

Re-admission of 3.5 mM Ca^2+^ to 0Ca containing PE (Fig. 2C) caused again a parallel time-dependent increase of [Ca^2+^]_i_ and tension to values of 0.18±0.02 RU and 0.78±0.11 mN/mm. When the SR Ca^2+^ release and corresponding force development (Fig. 2B) as well as the Ca^2+^ influx signal and corresponding contraction (Fig. 2C) were pair-wise summated, Ca^2+^ and tension signals as shown in Fig. 2D were very similar to the PE responses in control.

These results confirmed that the Ca^2+^ signals induced by 1 μM PE in normal KR solution consisted of an initial release of Ca^2+^ from internal stores and a subsequent influx of extracellular Ca^2+^ and that both Ca^2+^ signals were paralleled by a temporally-related force signal [13].

### Analysis of the PE-elicited contraction

#### L-type Ca^2+^ channels

**Fig 3:**
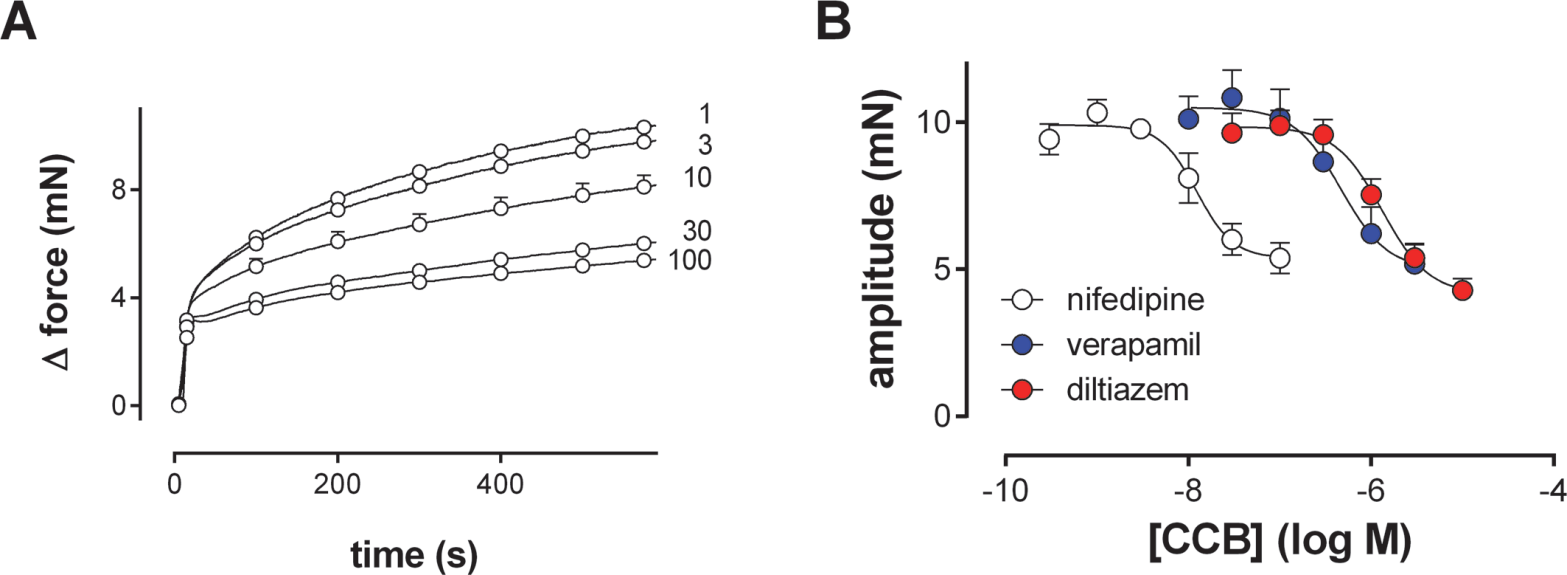
Effects of VGCC blockers on isometric contractions by PE. Nifedipine, verapamil and diltiazem partly inhibit isometric contractions induced by 1 μM PE in organ bath-mounted aortic segments. A) Contractions induced by 1 μM PE after incubating the segments with 1 to 100 nM nifedipine (n = 4); B) Ca^2+^ channel blocker (CCB) concentration-response curves for the inhibition of contractions induced by 1 μM PE.

Nifedipine (1–100 nM), verapamil (30–3000 nM) or diltiazem (0.1–10 μM), as representatives of the different classes of L-type Ca^2+^ channel blockers, did not reduce the fast phase of the PE (1 μM)-induced contraction (Fig. 3A), but effectively reduced the slow phase of contraction. Nifedipine inhibited 49±3% of the steady-state contraction with an IC_50_ of −7.94±0.07 −logM, diltiazem 59±3% with IC_50_ of −5.90±0.04 −logM and verapamil 57±5% with an IC_50_ of −6.31±0.10 −logM. These results again confirm that the fast phase of contraction is not due to extracellular Ca^2+^ influx via L-type Ca^2+^ channels, but most probably to intracellular Ca^2+^ release from the SR. The slow phase of the PE-contraction can be attributed for 50 to 60% to L-type Ca^2+^ influx of for 40 to 50% to Ca^2+^ influx via non-L-type Ca^2+^ channels.

#### Non-selective cation channels (NSCC)

**Fig 4:**
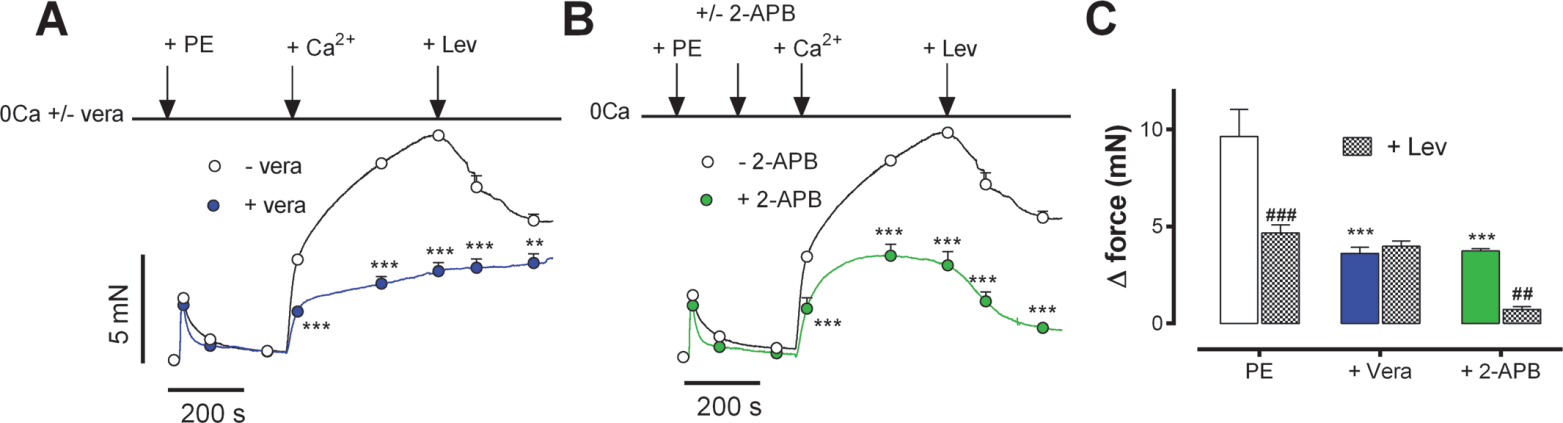
VGCC and NSCC contribute to the Ca^2+^ influx-mediated contraction elicited by PE. The PE-induced contraction following addition of 3.5 mM Ca^2+^ to 0Ca (n = 4) in the absence (black) and presence of 3 μM verapamil (Vera, blue) and B) in the presence of 100 μM 2-APB (green). Verapamil and 2-APB were added before and after the transient contraction by PE respectively. After 600 s, 100 nM levcromakalim (Lev) was added in each condition. C) Mean maximal contractions in control (PE), 3 μM verapamil (+Vera) and 100 μM 2-APB (+2-APB) in the absence (left bars) and presence (+ Lev, hatched bars) of 300 nM levcromakalim. ***: P<0.001, versus PE; ##, ###: P<0.01 and 0.001, + Lev versus control

The contribution of Ca^2+^ influx via L-type and non-L-type Ca^2+^ channels to the contraction by PE was further investigated by incubation of the segments with the L-type Ca^2+^ channel blocker verapamil and/or the putative non-selective cation channel blocker 2-aminoethoxydiphenyl borate (2-APB) [23]. In 0Ca^2+^, 3 μM verapamil did not affect the PE-induced phasic contraction, but significantly reduced the +Ca contraction (Fig. 4A). At 600 s, the contraction was inhibited by 59±10% (Fig. 4C). Because 2-APB inhibited the transient isometric contraction by PE in the absence of external Ca^2+^ in line with its capacity to block the SR IP_3_ receptor [23] (see also S1 Fig., S1 Text), it was applied after this transient contraction. The +Ca contraction in the presence of 100 μM 2-APB typically increased to a maximum at 200 to 250 s and then decreased again (Fig. 4B). At 600 s, 100 μM 2-APB suppressed the +Ca contraction by 59±6%. Finally, the combination of verapamil and 2-APB inhibited the +Ca contraction completely, indicating that the contraction remaining in the presence of a maximally effective concentration of 2-APB was due to L-type Ca^2+^ influx and the contraction remaining in the presence of a maximal dose of verapamil was caused by Ca^2+^ influx via non-selective cation channels.

**Fig 5:**
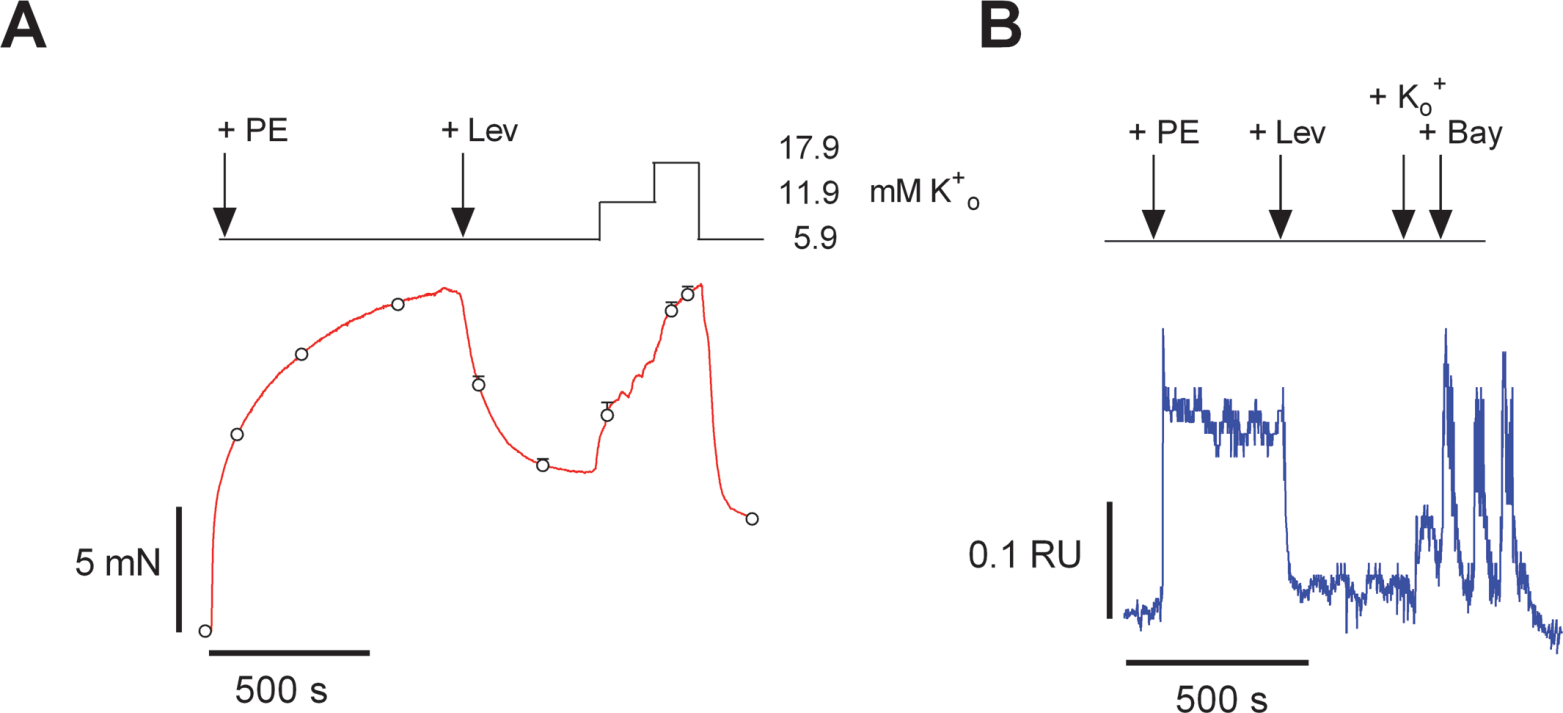
Modulation of the PE-induced contraction by window VGCC Ca^2+^ influx. A) The relaxation of the isometric pre-contraction (1 μM PE, red, organ bath) with addition of 300 nM levcromakalim (+ Lev) is reversed by increasing extracellular K^+^ (K_o_^+^) to 11.9 and 17.9 mM (n = 4). B) Representative example of a myograph experiment, showing the increase of intracellular Ca^2+^ (blue) with 1 μM PE (+ PE), decrease with 300 nM levcromakalim (+ Lev) and increase with supplementary addition of 10 mM external K^+^ (+ K_o_^+^) in combination with 30 nM BAY K8644 (+ Bay).

Levcromakalim, an ATP-dependent K^+^ channel agonist, hyperpolarises the VSMC V_m_ to the K^+^-equilibrium potential (E_K_: −80 to −85 mV) and decreases window Ca^2+^ influx [9]. Applied on top of the PE contraction, levcromakalim relaxed PE-induced contractions (Figs. 4 and 5A) and decreased intracellular Ca^2+^ (Fig. 5B). Remarkably, levcromakalim did not affect the contraction in the presence of 3 μM verapamil, but significantly reduced the contraction in the presence of 100 μM 2-APB by 81±4% (Fig. 4B). The inhibition of Ca^2+^ influx and contraction could be completely reversed by adding 50 μM glibenclamide (inhibitor of K^+^(ATP) channels, data not shown), by increasing extracellular K^+^ from 5.9 to 12 and 18 mM (Fig. 5A) or, as shown for intracellular Ca^2+^, by combining K^+^ increase with the L-type Ca^2+^ channel agonist BAY K8644 (Fig. 5B). These results suggest that the contraction in the presence of 2-APB is voltage-dependent and most probably due to L-type Ca^2+^ influx within the voltage range of its window.

#### Ca^2+^ influx-mediated contraction and resting membrane potential

In basal, non-stimulated conditions, there is a time-independent, baseline Ca^2+^ influx via L-type Ca^2+^ channels, which van be inhibited by removal of extracellular Ca^2+^, by hyperpolarization with levcromakalim or by L-type Ca^2+^ channel blockers [9,10]. Hence, if PE induces depolarisation and opening of L-type Ca^2+^ channels [15–19], the PE-elicited contraction of VSMC is expected to depend on shifts of V_m_ in the hyper- or depolarizing direction.

**Fig 6:**
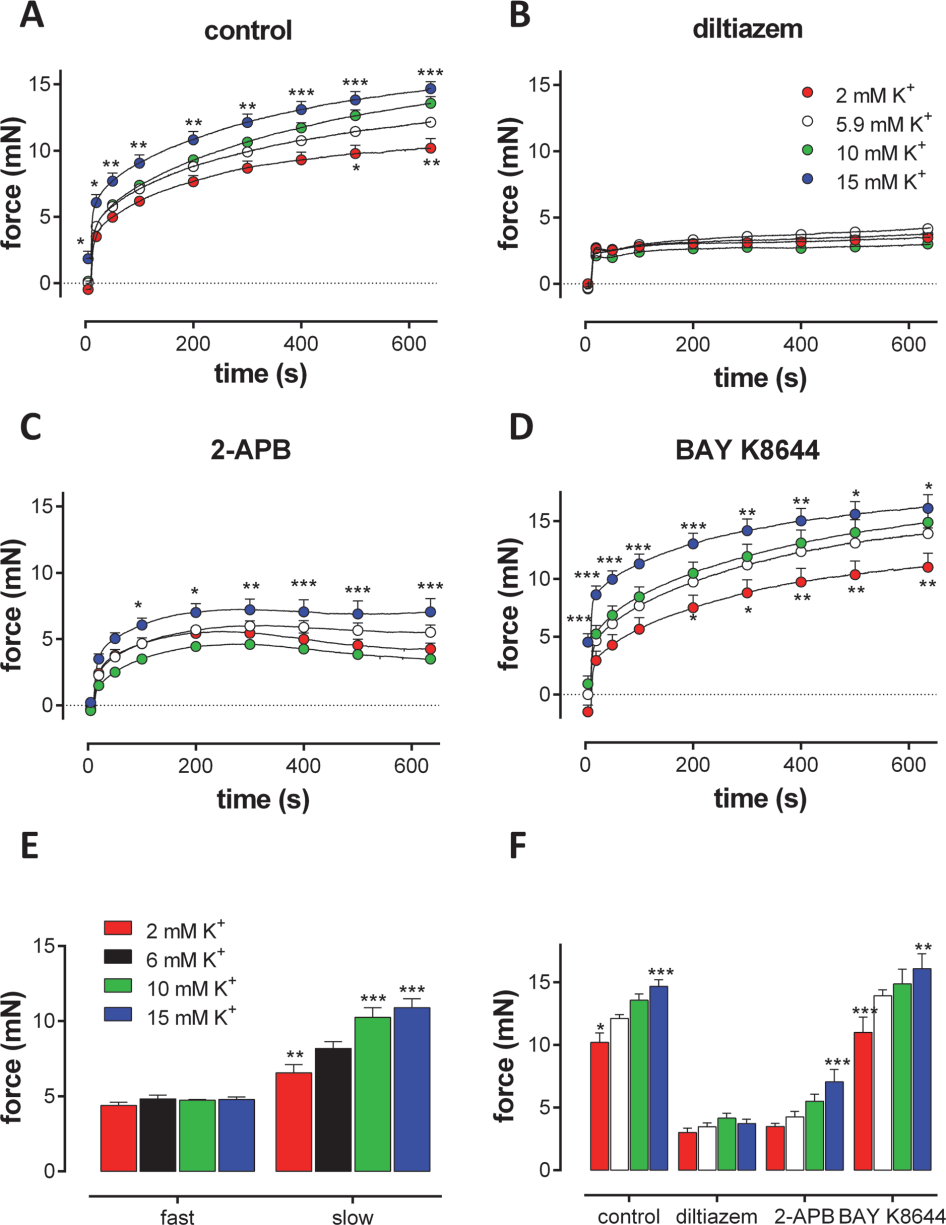
External K^+^- modulation of the PE-induced contraction. Isometric contractions induced by 1 μM PE at different extracellular K^+^ concentrations in the organ bath. Contractions were measured at 2, 5.9, 10 and 15 mM extracellular K^+^ in control (A) and in the presence of 35 μM diltiazem (B), 100 μM 2-APB (C) or 30 nM BAY K8644 (D). Bi-exponential analysis of the force development by PE revealed the amplitudes of fast and slow force components in control (E). The “steady-state” tonic contractions at 650 s in the absence (control) or presence of diltiazem, 2-APB or BAY K8644 are summarized in (F). *, **, ***: P<0.05, 0.01, 0.001 versus 5.9 mM K^+^ (n = 4–5).

Hyper- or depolarization of V_m_ of VSMC, by changing the external K^+^ concentration from 2 to 15 mM, may promote or inhibit the window Ca^2+^ influx [9,24] and the PE-elicited contraction. It was observed that not only the PE-induced isometric force but also the basal force was K^+^-dependent (-0.45±0.18 mN at 2 mM K^+^; +0.14±0.20 mN at 10 mM K^+^ and +1.85±0.58 mN at 15 mM K^+^). PE-induced isometric contraction at the different K^+^ concentrations displayed a bi-exponential time course (Fig. 6A, see also Fig. 1). Whereas the fast contraction phase was K^+^-independent, the slow phase changed with extracellular K^+^ and, hence, was voltage-dependent (Fig. 6E). The slow phase amplitude increased significantly from 84±6% at 2 mM K^+^ to 112±5% and 121±6% at 10 and 15 mM K^+^ with respect to 5.9 mM K^+^.

As was shown in the levcromakalim experiments, the isometric contraction of PE following inhibition of the L-type Ca^2+^ influx with 30 μM diltiazem was not dependent on the extracellular K^+^ concentration (Fig. 6 B, F). However, in the presence of 100 μM 2-APB, the tonic contraction increased with the external K^+^ concentration (Fig. 6C, F). Finally, when the L-type Ca^2+^ influx was stimulated with 30 nM BAY K8644, an agonist of L-type Ca^2+^ channels, the tonic PE contraction showed a similar K^+^-dependency as in control or 2-APB conditions (Fig. 6D, F).

#### Properties of non-selective cation channels (NSCC)

**Fig 7:**
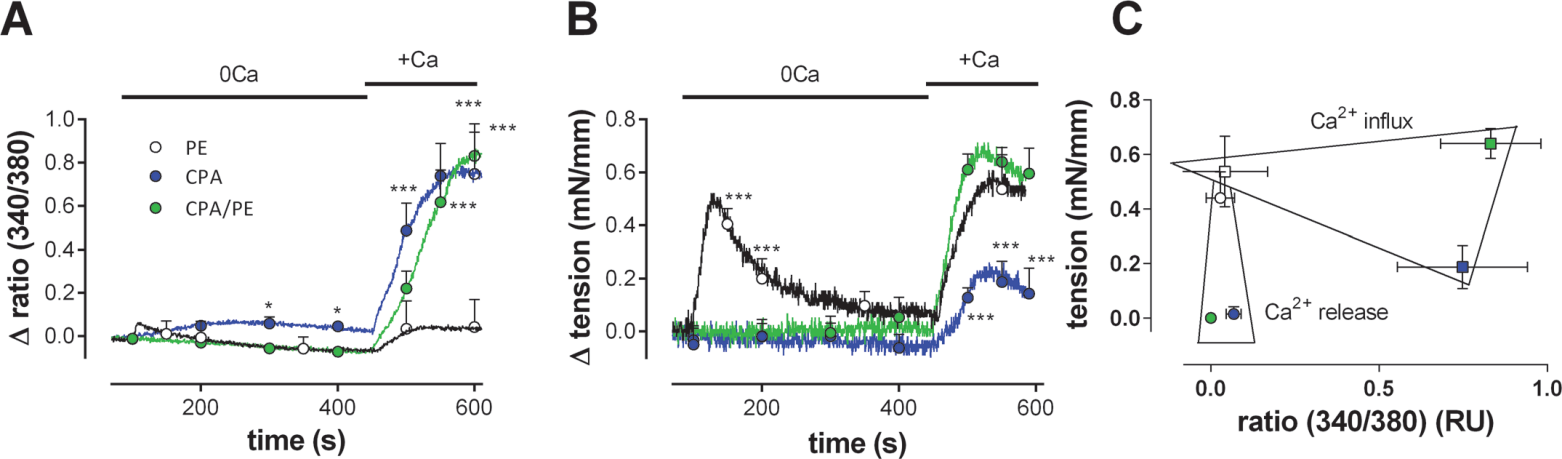
CPA and PE release intracellular Ca^2+^, cause Ca^2+^ influx and produce force by different mechanisms. Intracellular Ca^2+^ (A) and accompanying tension (B) were measured in aortic segments (n = 6) in the absence (0Ca) and after re-addition of 3.5 mM Ca^2+^ (+Ca) in the presence of 1 μM PE alone (black), in the presence of 10 μM CPA alone (blue) or in the presence of the combination (green). C) Force-calcium graph for the data in A and B with the squares referring to the +Ca data (Ca^2+^ influx) and the circles to the 0Ca data (Ca^2+^ release). *, ***: P<0.05, 0.001 CPA or CPA/PE versus PE

Ca^2+^ influx via non-selective cation channels could be increased by emptying CPA-sensitive Ca^2+^ stores before inducing PE-induced contractions. In aortic segments, 10 μM CPA causes inhibition of the CPA-sensitive Ca^2+^ store Ca^2+^ pump [25] and leads to a large increase of intracellular Ca^2+^ in the VSMC (Fig. 7A). When VSMC intracellular Ca^2+^ and isometric contraction were measured simultaneously, it was observed that in the absence of extracellular Ca^2+^, CPA induced a small and slow transient increase of intracellular Ca^2+^, which was not accompanied by an increase of force (Fig. 7B). In contrast, 1 μM PE caused a small and rapid transient increase of internal Ca^2+^, which elicited a substantial phasic tension. Re-addition of Ca^2+^ (+Ca) caused a large influx of Ca^2+^ from the extracellular medium for CPA alone when compared with PE alone but this large Ca^2+^ influx was accompanied by a significantly smaller increase in force than by PE. Finally, even this large increase of intracellular Ca^2+^, could in the presence of 1 μM PE, not substantially increase the force induced by PE alone. As summarized in the calcium-force graph of Fig. 7C, CPA mainly causes Ca^2+^ release and influx without majorly affecting force, whereas Ca^2+^ release and influx by PE causes a significant temporally-related increase in force. When CPA and PE were combined, both a large Ca^2+^ influx and a large force increase were simultaneously present, but not significantly larger than these of CPA (Ca^2+^) or PE (tension) alone.

**Fig 8:**
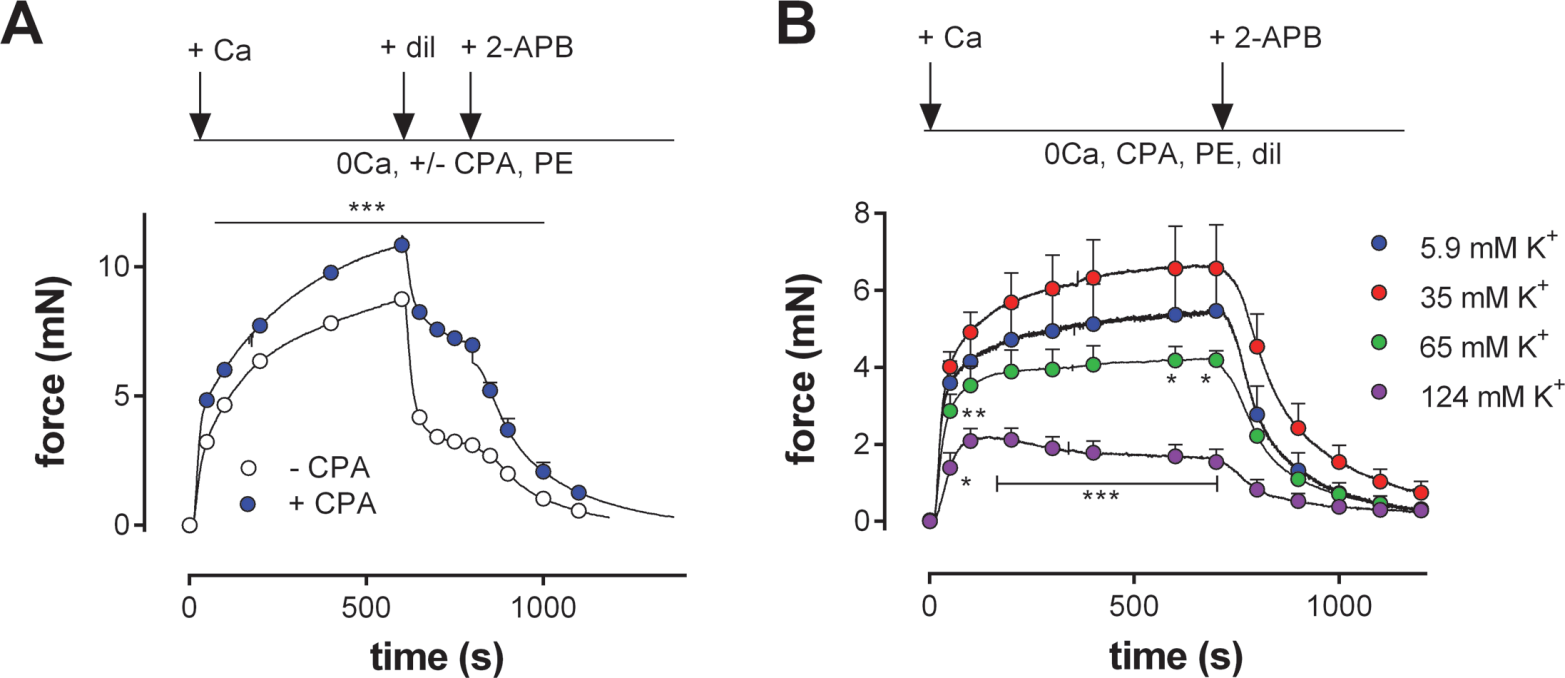
Modulation of the PE-induced contraction by NSCC Ca^2+^ influx. Isometric contractions induced by Ca^2+^ re-addition (+ Ca) to organ bath mounted aortic segments (n = 4) incubated in 0Ca in the presence of 1 μM PE A) with (blue) or without (white) 10 μM CPA (blue) or B) with 10 μM CPA and 35 μM dilitiazem (Dil) at 5.9 (blue), 35 (red), 65 (green) and 124 (purple) mM K^+^. In A) 35 μM diltiazem (+ dil) was added after 10 minutes to measure the relative amount of VGCC Ca^2+^ influx to the contractions. Finally, 50 μM 2-APB (+ 2-APB) was added to inhibit NSCC. ***: P<0.001 CPA versus control. In B) 50 μM 2-APB was added after 16 minutes to inhibit the contraction due to NSCC Ca^2+^ influx. *, **, ***: P<0.05, 0.01, 0.001 K^+^ versus control.

According to these data, CPA mobilizes Ca^2+^ from non-contractile Ca^2+^ stores and induces non-contractile Ca^2+^ influx. CPA did not significantly increase the tension induced by PE alone, but it empties Ca^2+^ stores, and thereby may affect the contraction induced by PE. Fig. 8A shows isometric contractions induced by 1 μM PE following addition of extracellular Ca^2+^ (control) when segments were first incubated with 0Ca + 10 μM CPA + 1μM PE to empty CPA-sensitive Ca^2+^ stores completely. In this condition, contraction increased to 124±2% (P<0.001, n = 4) of the contraction in the presence of PE alone. Diltiazem (35 μM) inhibited this contraction by 65±1% in the absence and by 36±2% (P<0.01,) in the presence of 10 μM CPA. Although CPA caused the mean contraction by PE to increase with about 24%, the contraction changed from mainly VGCC-mediated to mainly NSCC-mediated. Combining diltiazem with 50 μM 2-APB inhibited the contraction completely, suggesting that in the presence of CPA the relative contribution of NSCC was significantly increased from 35 to 64%.

**Fig 9:**
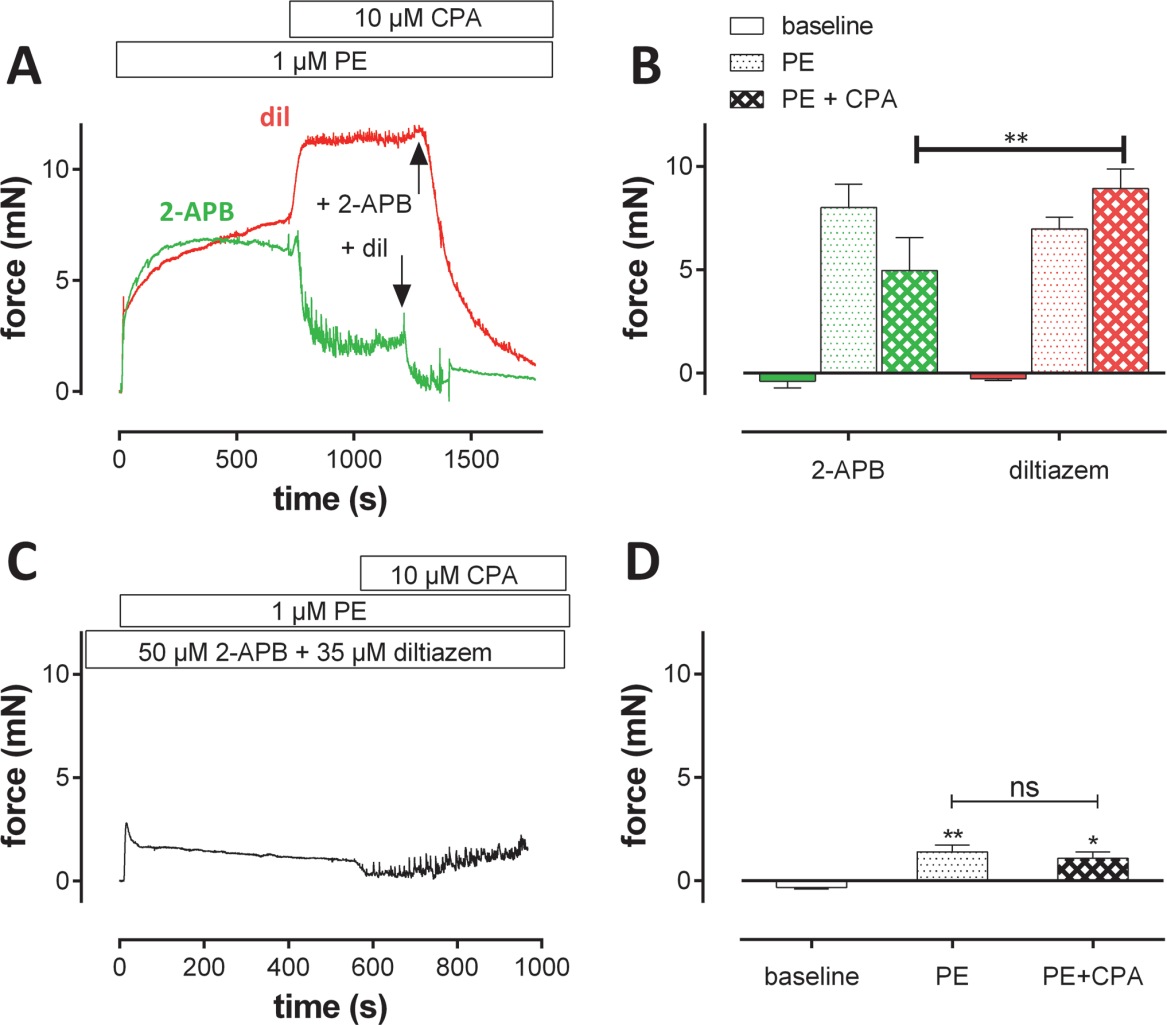
Effects of intracellular Ca^2+^ increase by CPA on contractions by PE in the presence of diltiazem or 2-APB. A) Representative example of PE-induced contractions after incubation of a segment with 35 μM diltiazem (red) or 50 μM 2-APB (green). 10 μM CPA was added in both conditions as indicated, after which 50 μM 2-APB (arrow, +2-APB)was added to the diltiazem condition and diltiazem (arrow, +dil) to the 2-APB condition. Figure B summarizes the results (n = 5). In C, the segments were incubated with 50 μM 2-APB and 35 μM diltiazem before the challenge with 1 μM PE. Subsequent addition of 10 μM CPA did not increase the PE-induced contraction. Part D summarizes the results with the change in isometric force by the diltiazem/2-APB in baseline conditions or following addition of 1 μM PE alone (PE) or in combination with 10 μM CPA (PE+CPA) (n = 4).*, **: p<0.05, 0.01 2-APB versus diltiazem condition in B or PE/PE+CPA versus baseline in D.

The large contribution of NSCCs to the contraction by PE in the presence of CPA is further illustrated in Fig. 8B, in which the voltage-dependent properties of the NSCC-mediated PE-induced contractions were investigated in more detail. Previous results suggested that the contraction by NSCC Ca^2+^ influx was nearly voltage-independent for K^+^ concentrations between 2 and 15 mM. Because NSCC have their reversal potential around 0 mV, contractions by 1 μM PE were measured at more depolarized membrane potentials. These were calculated to be −66 mV at 5.9 mM K^+^, −34 mV at 35 mM K^+^, −19 mV at 65 mM K^+^ and −3 mV at 124 mM K^+^ [9]. Segments were incubated with 0Ca, 10 μM CPA, 35 μM diltiazem and 1 μM PE at these extracellular K^+^ concentrations. Thereby, 35 μM diltiazem prevented the activation of L-type Ca^2+^ influx upon re-addition of extracellular Ca^2+^ to the depolarized segments. Moreover, the relative contribution of NSCC was increased by emptying CPA-sensitive Ca^2+^ stores (see Fig. 8A). Hence, the isometric contractions shown in Fig. 8B, are only due to Ca^2+^ influx via NSCC and, as such, could be completely inhibited by 50 μM 2-APB. The data show a voltage-dependent reduction in the isometric NSCC-mediated contraction at 65 mM K^+^ and 124 mM K^+^, suggestive for NSCC activation in the presence of PE. That CPA directly stimulates NSCC is illustrated in Figs. 9A and B. Isometric contractions were elicited by 1 μM PE in the presence of 35 μM diltiazem or 50 μM 2-APB, after which 10 μM CPA was added. The contraction in the presence of diltiazem, which is attributed to NSCC Ca^2+^ influx, was increased by 10 μM CPA. This contraction was completely inhibited by 50 μM 2-APB. On the other hand, the PE-induced contraction after incubation of the aortic segments with 2-APB, is mainly due to VGCC Ca^2+^ influx and is significantly decreased by 10 μM CPA. This attenuated contraction was inhibited with 35 μM diltiazem. When the segments were incubated with a combination of 50 μM 2-APB with 35 μM diltiazem, 1 μM PE caused a transient contraction, which was not increased upon subsequent addition of 10 μM CPA (Figs. 9C and D).

## Discussion

In this study, we show that isometric contractions of mouse aortic segments by α_1_-adrenoceptor stimulation with PE involve complex interactions between Ca^2+^ release from SR Ca^2+^ stores and concomitant activation of Ca^2+^ influx via VGCC and NSCC. The biphasic contractions display a fast and transient component, which corresponds to the intracellular Ca^2+^ release from the SR. The slow and sustained component is attributed to Ca^2+^ influx from the extracellular medium and determines the steady-state contraction.

Hyperpolarization, moderate depolarization or pharmacological VGCC stimulation affect the slow but not the fast force component of the PE-induced contraction, especially after inhibition of NSCC. This is in line with the properties of window L-type Ca^2+^ influx via VGCC. PE-induced contractions due to Ca^2+^ influx via NSCC are activated by causing Ca^2+^ release from non-contractile Ca^2+^ stores.

### Fast component of PE-elicited contraction

**Fig 10:**
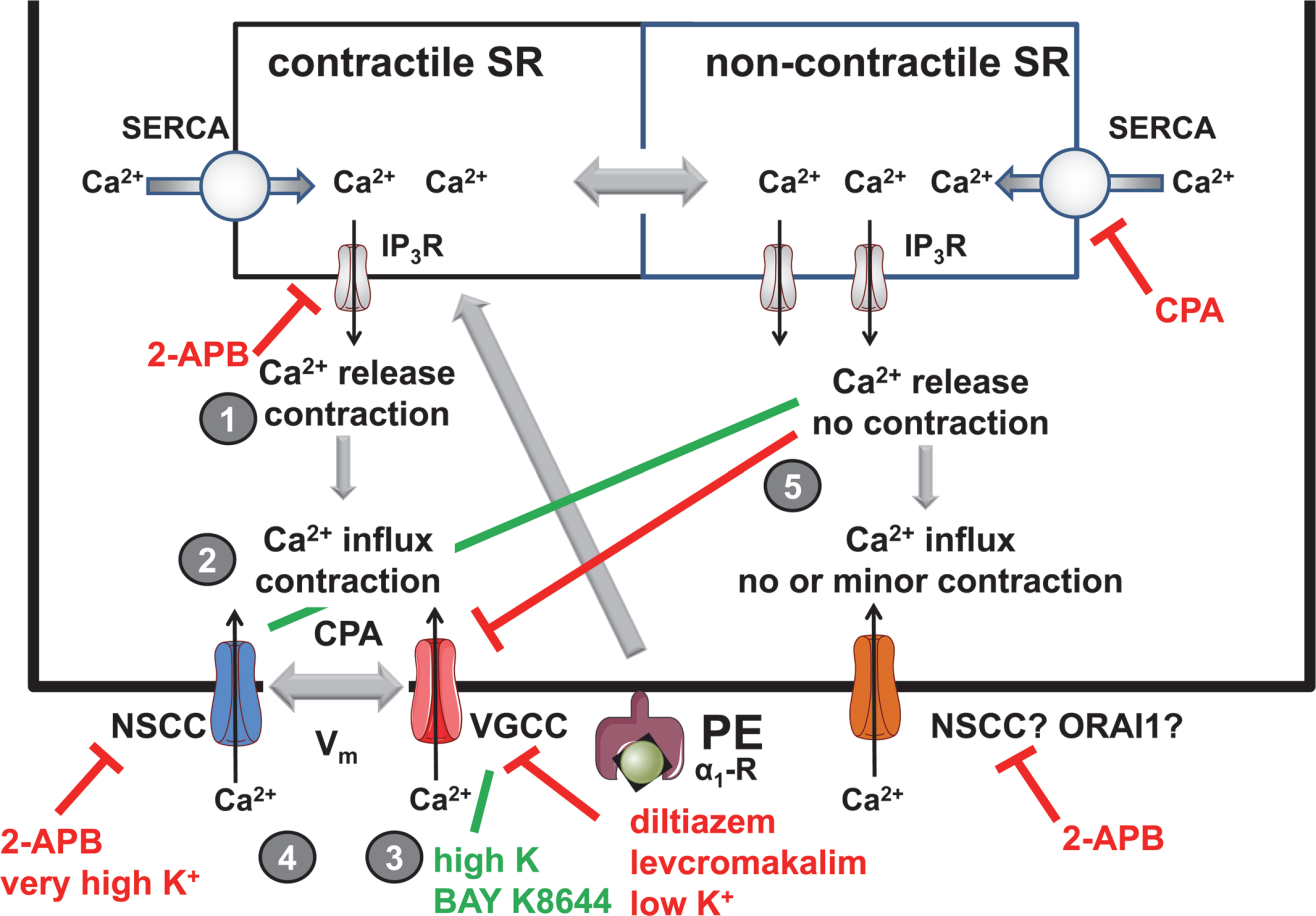
Scheme showing the Ca^2+^ mediated processes involved in α_1_-adrenoceptor stimulation of mouse aortic segments with PE. PE causes phasic Ca^2+^ increase and concomitant contraction by releasing Ca^2+^ from the SR (event 1). This is accompanied by influx of Ca^2+^ via complex interactions between NSCC and VGCC and the steady-state contraction by PE is determined by the relative contribution of window Ca^2+^ influx via VGCC (very voltage-dependent) and Ca^2+^ influx via NSCC (less voltage-dependent) (event 2). Window VGCC Ca^2^ influx and related contraction are inhibited by diltiazem, membrane potential repolarization with K^+^ or levcromakalim and Ca^2+^ release from non-contractile Ca^2+^ stores by CPA and stimulated by high K^+^ and BAY K844 (event 3). NSCC Ca^2^ influx and related contraction are inhibited by 2-APB and very high K^+^ (strong depolarization to −20 mV or less negative) and stimulated by Ca^2+^ release from non-contractile Ca^2+^ stores with CPA (event 4). CPA causes high Ca^2+^ release from a non-contractile compartment of the SR (event 5). Emptying of the non-contractile Ca^2+^ store with CPA causes large Ca^2+^ influx, which is accompanied with minor contraction in the absence of PE, but which turns the PE-induced contraction to one that is mainly mediated by NSCC. This points to a complex interaction between the non-contractile and contractile SR Ca^2+^ stores and their refilling via VGCC and/or different NSCC.

As demonstrated for depolarization with extracellular K^+^ elevation [9,10], PE-induced contractions displayed a bi-exponential time course with a fast and slow force component. Contrary to K^+^-depolarized segments, where the fast, transient component corresponded to a population of L-type Ca^2+^ channels that quickly activated and completely inactivated [9], the fast and transient component of the PE-response was voltage-independent, independent of external Ca^2+^, could not be inhibited with L-type Ca^2+^ channel blockers, but was sensitive to 2-APB. Experiments in the absence of external Ca^2+^ further indicated that the fast force component was temporally related to an increase of intracellular Ca^2+^. This component probably corresponds to Ca^2+^ release from contractile intracellular Ca^2+^ stores via IP_3_-sensitive receptors (Fig. 10, event 1) [15].

### Slow component of PE-elicited contraction

According to the kinetic and pharmacological analysis of PE-induced force development in mouse aorta, the slow component contributed for 60% to the total contraction. In rat aorta, this was about 25% [22], indicating that the store-dependent Ca^2+^ release and concomitant contraction is far more important in rat than in mouse aorta. From our experiments, however, it was clear that the slow force development, together with Ca^2+^ sensitizing mechanisms, determined the steady-state force. Hence, the relative contribution of fast and slow components to the total contraction provides information only on the amount of store-dependent Ca^2+^ release with respect to Ca^2+^ influx, but not on the steady-state contraction.

Force by high K^+^-induced depolarization of mouse aortic segments [9] or by α_1_-adrenergic stimulation of rat renal artery [26] was completely inhibited by L-type Ca^2+^ channel blockers, suggesting that these contractions were completely due to VGCC Ca^2+^ influx. PE-induced contraction in mouse aortic segments was, however, only for 50 (nifedipine) to 60% (verapamil, diltiazem) inhibited by VGCC blockers, which indicates the occurrence of a non-L-type Ca^2+^ influx. The tonic component of PE-contracted segments was temporally related to a Ca^2+^ signal resulting from the influx of Ca^2+^ via different Ca^2+^ permeable ion channels (Fig. 10, event 2). Similar results were obtained in penile small arteries of rat, where α_1_-adrenergic constriction with PE also involved Ca^2+^ entry via both L-type (50%) and 2-APB (50%)-sensitive receptor-operated channels, as well as Ca^2+^ sensitization mechanisms [27]. Of the four mechanisms at the basis of PE-induced contraction in rat tail artery, i.e. 1) depolarization and Ca^2+^ influx via VGCC; 2) V_m_-independent Ca^2+^ influx probably via direct activation of VGCC; 3) increased Ca^2+^ sensitivity of the contractile elements and 4) release of Ca^2+^ from intracellular stores [28], the second mechanism was different in mouse aortic segments. The “voltage-independent” component occurred in the presence of VGCC blockers and could be inhibited with 2-APB, suggesting that it was mediated by NSCC permeable to Ca^2+^ [23]. One should, however, be cautious in interpreting the 2-APB data, because 2-APB has been described to block also VGCC (see S1 Fig., S1 Text) [29,30]. Nevertheless, the observations that 1) 2-APB completely inhibited the PE-induced contraction after eliminating the L-type Ca^2+^ influx with diltiazem and that 2) the contraction remaining in the presence of diltiazem was voltage-independent within a small voltage range or did not change with addition of levcromakalim, suggest that PE elicits Ca^2+^ influx via NSCC.

At physiological V_m_ or physiological K^+^ concentrations below 20 mM [31], the slow component of the contraction by PE increased gradually and significantly with small elevations of K^+^ or decreased with hyperpolarization induced by the ATP-dependent K^+^ channel opener levcromakalim (Fig. 10, event 3). Our results indicate that a small depolarization as with elevated K^+^ or a hyperpolarization as with lower K^+^ or with levcromakalim affects PE contraction because of an increase or decrease of the background window Ca^2+^ influx. This might be important in pathophysiological conditions such as hypertension or endothelial dysfunction where it has been described that VSMC are depolarised [24]. For example, mechanical stretch has been described to cause increase of intracellular Ca^2+^ via stretch-activated NSCC, depolarization and VGCC activation and these events may be amplified in hypertension [32–34].

CPA, an inhibitor of SERCA, causes intracellular Ca^2+^ increase without eliciting associated contractile responses. This may be due to the absence of a myofilament sensitizing effect of CPA, but can also be explained by assuming that the CPA-sensitive SERCA pump was predominantly expressed in the non-contractile SR Ca^2+^ store [25,35]. In mouse aortic segments, we confirmed that the intracellular VSMC Ca^2+^ elevation by SERCA inhibition with CPA, caused no or only small contractions (Fig. 10, event 4). Remarkably, although the contractile effect of CPA was negligible and the combination with PE did not or did only slightly increase the contractile effect of PE, it transformed the mainly VGCC-mediated PE-induced contraction to a mainly NSCC-mediated contraction. The PE-induced contraction mediated by VGCC Ca^2+^ influx, hence in the presence of 50 μM 2-APB, was not increased but inhibited by 10 μM CPA. On the other hand, the PE-elicited contraction mediated by NSCC Ca^2+^ influx, hence in the presence of 35 μM diltiazem, was stimulated by CPA. These data do not support the hypothesis that Ca^2+^ increase by CPA, similarly to PE, increases sensitivity of the myofilaments to Ca^2+^. They suggest complex interactions between contractile and non-contractile SR Ca^2+^ stores and store-dependent influx of Ca^2+^ via ion channels (Fig. 10, event 3). Because these interactions might have important consequences for the re-filling of both Ca^2+^ stores via store-operated Ca^2+^ permeable ion channels and phenotypic switching of VSMC in pathological conditions [36,37], they need further investigation.

At 124 mM K^+^ the estimated V_m_ is close to 0 mV, which is the reversal potential for NSSC. Nevertheless, there was still a substantial contraction, suggesting that the reversal potential of the NSCC leading to the isometric contraction at 124 mM K^+^ displays preferential Ca^2+^ above K^+^ selectivity. The low voltage-dependency at moderate K^+^ concentrations is suggestive for outward rectification of the NSCC current involved in α_1_-adrenoceptor stimulation with PE. It should be mentioned, however, that changes of internal pH by exchange of Na^+^ for K^+^ ions in the high K^+^ solution and partial inhibition of the Na^+^-dependent acid extruders such as the Na^+^/H^+^ exchanger or the Na^+^/HCO_3_^-^ transporter, may also affect isometric contractions in conditions of low external Na^+^.

In conclusion, α_1_-adrenergic stimulation of mouse aortic segments causes transient contraction because of IP_3_-mediated Ca^2+^ release from the SR and concomitant tonic contraction due to Ca^2+^ influx via VGCC and NSCC. At physiological V_m_ (physiological K^+^), the baseline and PE-induced force are voltage-dependent mainly because V_m_ of VSMC resides in the window voltage range of the VGCC. Hyperpolarization of V_m_ with levcromakalim or low K^+^ avoids or diminishes activation of window Ca^2+^ influx and causes contraction by Ca^2+^ influx mainly via NSCC. By causing intracellular Ca^2+^ release from non-contractile Ca^2+^ stores with CPA, PE-induced contractions can be turned from mixed VGCC/NSCC-mediated to majorly NSCC-mediated. Results of this study emphasize the important role of VGCC and NSCC in mouse aortic performance and further indicate that although the contractile performance of aortic segments in different conditions may be the same, the relative contributions of VGCC or NSCC Ca^2+^ influx may significantly differ. The extreme voltage-dependent VGCC window contractions and the complex interactions with NSCC and non-contractile SR Ca^2+^ stores may have consequences for the development of arterial de-stiffening and antihypertensive therapies.

## Supporting Information

S1 Text (DOCX)

S1 Fig Inhibition of PE(1 μM)-mediated phasic (A) and tonic (B) contractions by 2-APB. A. Phasic contractions by 1 μM PE were measured 3 minutes after applying 0Ca. The concentration-response (area under the curve, AUC) curve in C revealed an IC_50_ of 34±4 μM 2-APB. B. Tonic contractions by 1 μM PE upon re-addition of 3.5 μm Ca^2+^ to the 0Ca solution containing 1 μM PE. The concentration-response (isometric force) curve in D revealed an IC_50_ of 38±5 μM and was not significantly different from the IC_50_ for inhibition of the tonic contraction. (n = 5)
(TIF)
