# Supplementary material for: Dissecting out the Complex Ca2+-Mediated Phenylephrine-Induced Contractions of Mouse Aortic Segments
Source: PLoS One. 2015 Mar 24;10(3):e0121634. doi: 10.1371/journal.pone.0121634 (PMC4372603; doi:10.1371/journal.pone.0121634)
Supplement: S1 Text — (DOCX) [file pone.0121634.s001.docx]

**Dissecting out the complex Ca^2+^-mediated phenylephrine-induced contractions of mouse aortic segments**

Paul Fransen^1^, Cor E. Van Hove^2^, Arthur J.A. Leloup^1^, Wim Martinet^1^, Guido R.Y. De Meyer^1^, Katrien Lemmens^2^, Hidde Bult^2^, Dorien M. Schrijvers^1^

^1^ Department of Pharmaceutical Sciences, University of Antwerp, Antwerp, Belgium

^2^ Department of Medicine and Health Sciences, University of Antwerp, Antwerp, Belgium

Running head:

Corresponding author: Paul Fransen, [paul.fransen@uantwerpen.be](mailto:paul.fransen@ua.ac.be), Laboratory of Physiopharmacology, Department Pharmaceutical Sciences, Campus Drie Eiken, Universiteitsplein 1, 2610 Antwerp

**Supplementary information**

**Effects of 2-APB on phasic and tonic contractions by PE in mouse aortic segments.**

The putative non-selective cation channel blocker, 2-APB, was used in the present study at concentrations of 50 or 100 µM in order to block non-selective cation channels (NSCC, store-operated calcium influx). 2-APB, however, also inhibited the PE-induced phasic contraction by blocking the IP_3_ receptor (figure S1 A) and subsequent Ca^2+^ influx (figure S1 B). This occurred with similar IC50-values indicating both processes are linked (figure S1 C, D). Therefore, and to study its effect on NSCC, 2-APB was always applied after eliciting the IP_3_-mediated contraction by PE or after inhibition of L-type Ca^2+^ channels with diltiazem or verapamil to avoid non-specific effects on L-type Ca^2+^ channels.


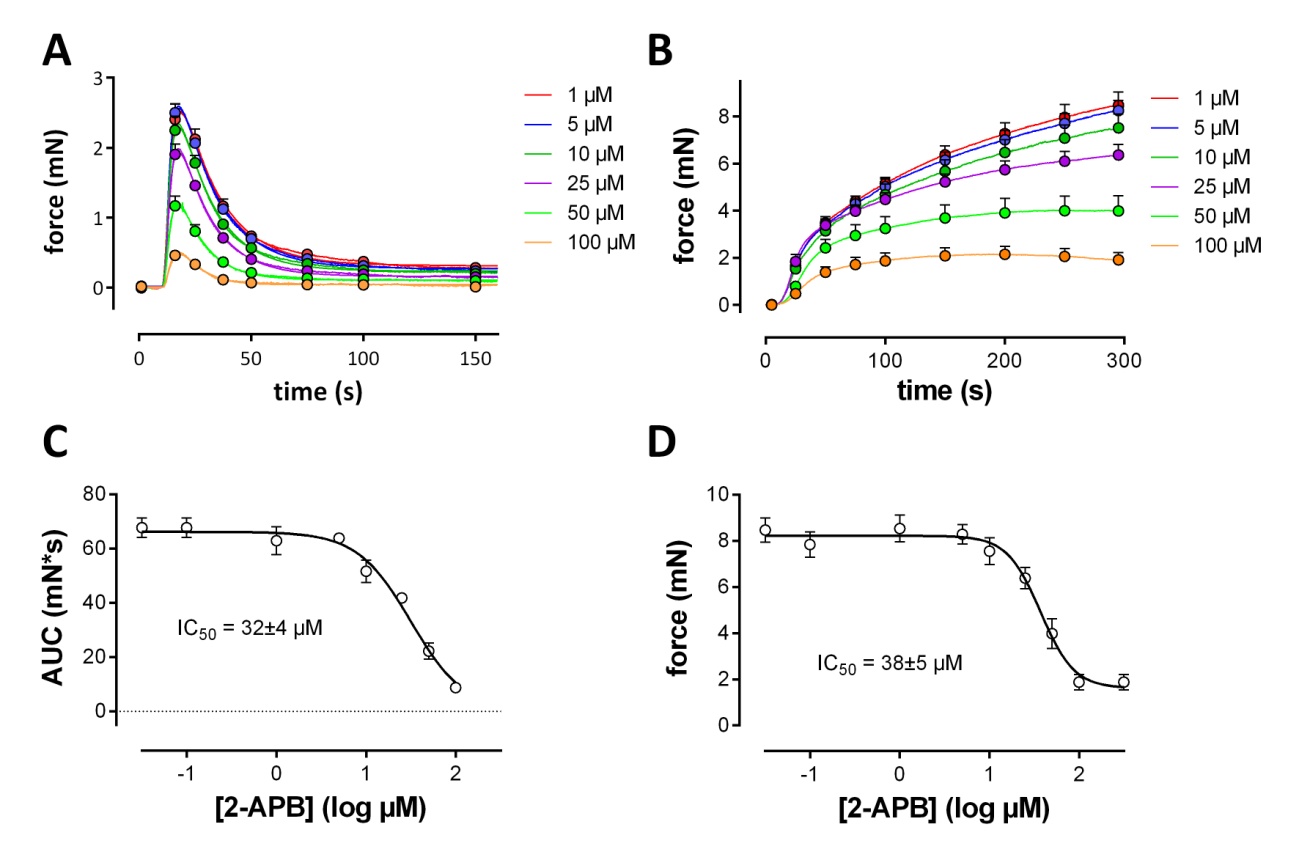


**S1. Fig 1. Inhibition of PE(1 µM)-mediated phasic (A) and tonic (B) contractions by 2-APB.** A. Phasic contractions by 1 µM PE were measured 3 minutes after applying 0Ca. The concentration-response (area under the curve, AUC) curve in C revealed an IC_50_ of 34±4 µM 2-APB. B. Tonic contractions by 1 µM PE upon re-addition of 3.5 µm Ca^2+^ to the 0Ca solution containing 1 µM PE. The concentration-response (isometric force) curve in D revealed an IC_50_ of 38±5 µM and was not significantly different from the IC_50_ for inhibition of the tonic contraction. (n=5)

**Figure Legends**

**S1 Fig 1. Inhibition of PE(1 µM)-mediated phasic (A) and tonic (B) contractions by 2-APB.** A. Phasic contractions by 1 µM PE were measured 3 minutes after applying 0Ca. The concentration-response (area under the curve, AUC) curve in C revealed an IC_50_ of 34±4 µM 2-APB. B. Tonic contractions by 1 µM PE upon re-addition of 3.5 µm Ca^2+^ to the 0Ca solution containing 1 µM PE. The concentration-response (isometric force) curve in D revealed an IC_50_ of 38±5 µM and was not significantly different from the IC_50_ for inhibition of the tonic contraction. (n=5)
