# Supplementary figures and images for: Dissecting out the Complex Ca2+-Mediated Phenylephrine-Induced Contractions of Mouse Aortic Segments
Source: PLoS One. 2015 Mar 24;10(3):e0121634. doi: 10.1371/journal.pone.0121634 (PMC4372603; doi:10.1371/journal.pone.0121634)

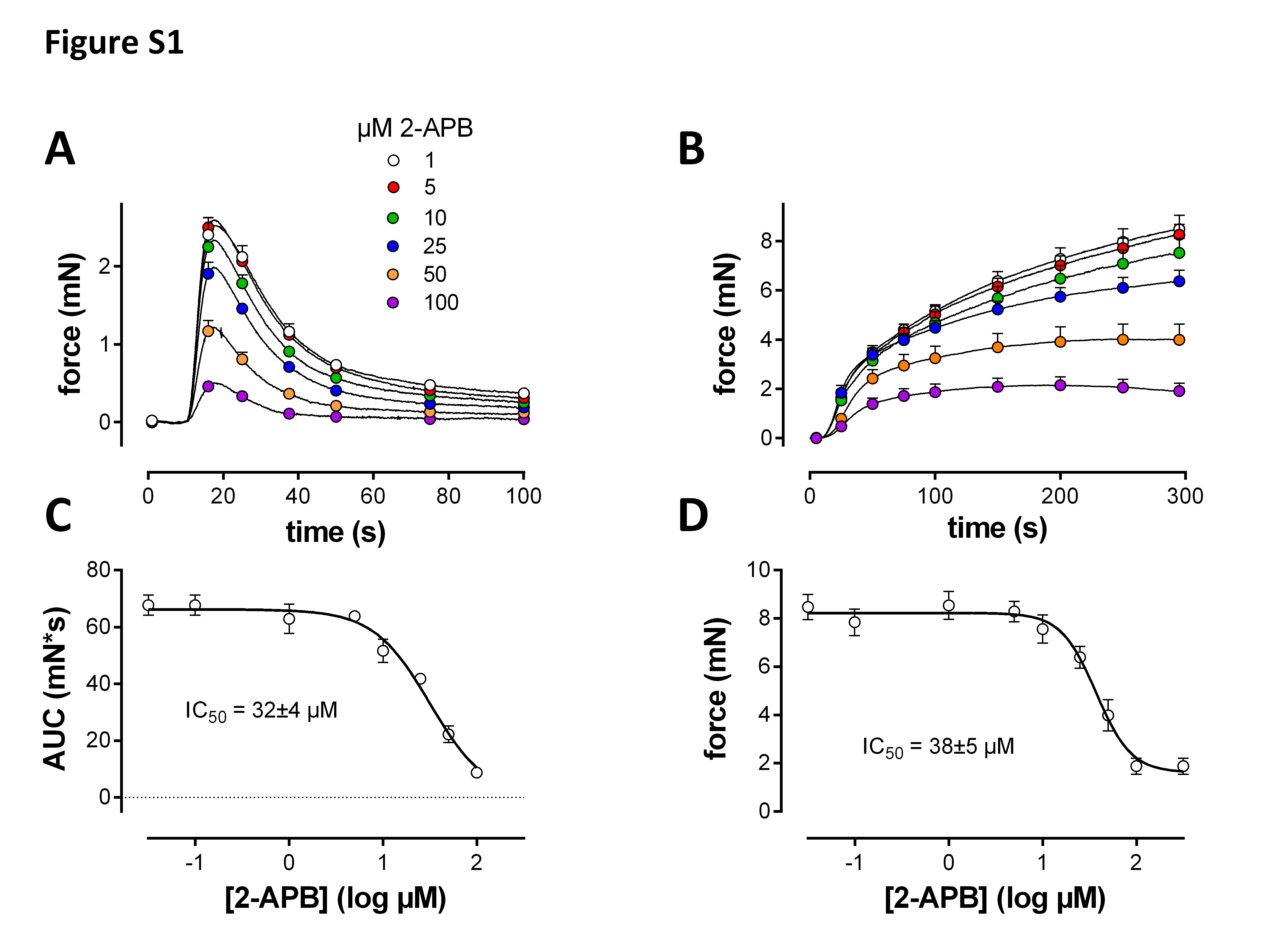

Supplement: S1 Fig — A. Phasic contractions by 1 μM PE were measured 3 minutes after applying 0Ca. The concentration-response (area under the curve, AUC) curve in C revealed an IC50 of 34±4 μM 2-APB. B. Tonic contractions by 1 μM PE upon re-addition of 3.5 μm Ca2+ to the 0Ca solution containing 1 μM PE. The concentration-response (isometric force) curve in D revealed an IC50 of 38±5 μM and was not significantly different from the IC50 for inhibition of the tonic contraction. (n = 5) (TIF) [file pone.0121634.s002.tif]
